# Discussing life expectancy with surgical patients: Do patients want to know and how should this information be delivered?

**DOI:** 10.1186/1472-6947-8-24

**Published:** 2008-06-15

**Authors:** Michael G Clarke, Katherine P Kennedy, Ruaraidh P MacDonagh

**Affiliations:** 1 Department of Urology & General Surgery, Taunton & Somerset Hospital, Musgrove Park, Taunton, TA1 5DA, UK

## Abstract

**Background:**

Predicted patient life expectancy (LE) and survival probability (SP), based on a patient's medical history, are important components of surgical decision-making and informed consent. The objective of this study was to assess patients' interpretation of and desire to know information relating to LE, in addition to establishing the most effective format for discussion.

**Methods:**

A cross sectional survey of 120 patients (mean age = 68.7 years, range 50–90 years), recruited from general urological and surgical outpatient clinics in one District General and one Teaching hospital in Southwest England (UK) was conducted. Patients were included irrespective of their current diagnosis or associated comorbidity. Hypothetical patient case scenarios were used to assess patients' desire to know LE and SP, in addition to their preferred presentation format.

**Results:**

58% of patients expressed a desire to know their LE and SP, if it were possible to calculate, with 36% not wishing to know either. Patients preferred a combination of numerical and pictorial formats in discussing LE and SP, with numerical, verbal and pictorial formats alone least preferred. 71% patients ranked the survival curve as either their first or second most preferred graph, with 76% rating facial figures their least preferred. No statistically significant difference was noted between sexes or educational backgrounds.

**Conclusion:**

A proportion of patients seem unwilling to discuss their LE and SP. This may relate to their current diagnosis, level of associated comorbidity or degree of understanding. However it is feasible that by providing this information in a range of presentation formats, greater engagement in the shared decision-making process can be encouraged.

## Background

Life expectancy (LE) can be defined as the average number of years an individual of a given age is expected to live if current mortality rates apply. It represents an important component in surgical decision-making, alongside disease parameters and patient choice. Indeed the issue of LE can make the difference between patients receiving treatment and being denied it; for example current UK guidelines recommend that patients with early prostate cancer should be offered curative treatment only if their estimated LE is more than 10 years [1].

An increasing amount is now known about the factors that influence patient LE, in addition to mortality and morbidity associated with surgical procedures. Indeed a multitude of comorbidity-related risk prediction tools are now used in planning patient treatment (e.g. POSSUM, ASA and CPEX testing) [2-4]. This has enabled surgeons to provide patients with a range of numerical and non-numerical prognostic data in order to facilitate informed consent. Successful 'shared decision making', however, relies on a surgeon's ability to present this evidence-based risk information in a clear and balanced format, in addition to considering patients' preferences and values [5].

Previous research has highlighted the effects of presentation, wording and 'framing' of risk information on both clinicians' and patients' interpretation of numerical data and its impact on treatment choice [6-11]. In consenting patients for surgical procedures, it is common for clinicians to use numerical risk information, based on both evidence-based research and regional audit. Whilst patients often perceive numerical data as more precise, their understanding and recall of this information may be inaccurate, acquiring only a 'gist' of the information as either a high or low risk – known as the 'fuzzy trace theory' [12,13]. Indeed a proportion of patients prefer verbal descriptions of risk (e.g. likely, almost certain) [14]. The additional use of graphical formats (e.g. survival curve, bar chart) have been used to help patients understand risk information relating to different treatment options [15,16]. However patients' preferences and accuracy of comprehension have varied depending on the clarity, accuracy and 'framing' associated with the graph.

Whilst much of the previous research has examined risk communication in relation to survival probability (SP) and intervention-associated risk/benefit, none have explicitly examined patients' interpretation of, or desire to know, information relating to their life expectancy (LE). This study therefore aimed to assess patients' understanding of information relating to hypothetical patient LE and SP, their preferred format for presentation and their desire to know this information if it were available.

## Methods

This study was approved by the Huntingdon Research Ethics Committee. All patients were receiving care in the general urological and surgical outpatient clinics of a District General Hospital and a Teaching hospital in Southwest England, UK. Patients aged 50–90 years, representing the commonest age group attending these clinics, with scheduled appointments were approached for study participation after being seen for their appointment. Patients were included irrespective of their current diagnosis or associated comorbidity. Informed consent was obtained from each patient prior to study participation.

All patients completed a questionnaire, containing five sections, in the presence of the same investigator (MC). The written information for each section was clarified by discussion with the investigator, to more closely resemble 'real life' consultations. Patients were given the opportunity to ask questions during this time.

### Section 1 – Patient characteristics

Patients provided demographic information relating to their age, sex and number of years in full time education.

### Section 2 – Numerical interpretation

To examine the understanding of survival probability and life expectancy, patients were presented with two statements relating to hypothetical scenarios: 'A person has a 70% chance of being alive in the next 10 years' and 'A 75 year old person has a predicted life expectancy of 10 years'. Patients answered five questions relating to each statement (e.g. this person has an equal chance of being dead or alive in ten years – yes or no), in addition to selecting a word from a list of 8 (e.g. poor, good, almost certain), which they felt best described the chance of the person being alive in 10 years in each scenario.

### Section 3 – Verbal interpretation

To examine the understanding of percentages, patients were required to place a mark on a 10 cm line marked out 0 – 100%, which they felt best described the percentage chance of a hypothetical patient being alive in 10 years based on 8 verbal descriptions (*Good*, *Somewhat unlikely*, *Poor*, *Probable*, *Almost certain*, *Almost impossible*, *Improbable *and *Somewhat likely*).

### Section 4 – Visual interpretation

**Figure 1:**
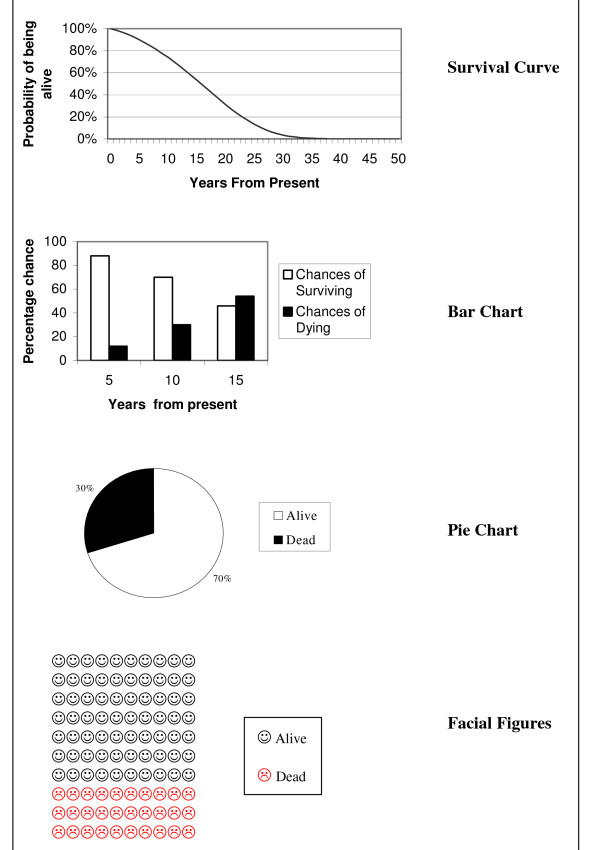
Four different graphical formats that patients were asked to rate relating to a person with a '70% chance of being alive in 10 years': Survival curve, bar chart, pie chart and facial figures.

**Figure 2:**
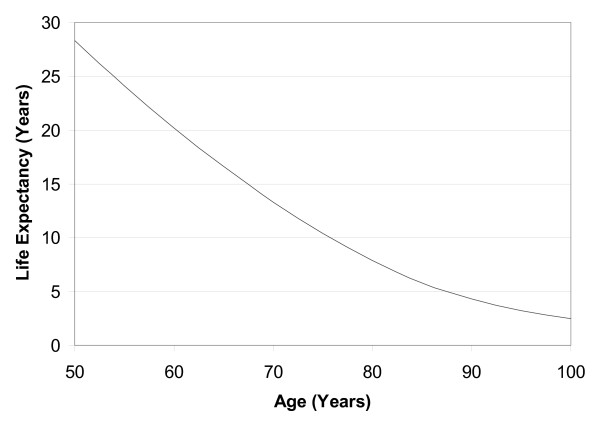
Line graph that patients were asked to rate displaying life expectancy.

To examine patients' preferences for graphical formats displaying survival probability, four examples which are most commonly used in the medical literature and public press were displayed: survival curve, bar chart, pie chart and facial figures (fig. 1). Each figure related to a hypothetical patient with a '70% chance of being alive in 10 years'. In addition a line graph displaying predicted LE for persons aged 50–100 years was displayed (fig. 2). Patients were asked to rate how much they liked each graph on a 5 cm Likert scale marked 1 to 5 (1 = not at all, 5 = extremely). In addition patients' reasons for their ratings were recorded as free text.

### Section 5 – Overall patient preferences

To examine patients' preferences for overall presentation format, they were asked to rank their top three choices of format from a list of six (*numbers only, words only, pictures only, numbers & words, numbers & pictures and words & pictures*). In addition patients were asked whether they would want to know their own predicted LE, SP or both, if they were available. Reasons for their decision were recorded as free text.

Statistical power calculation concluded that 120 patients would be sufficient to calculate a sample mean with appropriate confidence intervals. The inclusion of 60 males and 60 females would also enable statistical comparison between sexes using the Mann-Whitney statistical test. For all analyses a *p *value of ≤ 0.05 was taken to indicate statistical significance. Patients' interpretation of verbal risk information was assessed using a 10 cm visual analogue scale. Preferences for graphical risk presentation were assessed using a 5 cm Likert scale.

## Results

120 patients, 60 males and 60 females, participated. Their ages ranged from 50–90 years, with a mean of 68.7 years (69.4 males, 68.1 females). Eighty-eight patients (73.3%) completed full time education aged ≤ 16 years, 29 (24.2%) completed aged 18 years, 3 (2.5%) completed following a university undergraduate degree and none had undertaken a postgraduate degree.

### Section 2 – Numerical interpretation

For this section, assessing patients' interpretation of numerical statements relating to LE and survival probability, 24 patients (20%) answered all five questions correctly and 5 (4.2%) answered all incorrectly, with mean (SD) of 3.03 (± 1.45) questions answered correctly for the question relating to a '70% chance of being alive in 10 years'. 64 (53.3%) patients described this percentage as a *Good chance *of being alive in 10 years.

36 patients (30%) answered all five questions correctly and 0 answered all incorrectly, with a mean (SD) of 3.71 (± 1.09) questions answered correctly for the question relating to a '75 year old person has a predicted life expectancy of 10 years'. 58 (48.3%) patients described this as a *Good chance *of being alive in 10 years.

No statistically significant differences were seen between the sexes in the number of questions answered correctly for survival probability (p = 0.86) or life expectancy (p = 0.48); nor was there any significant difference between those leaving full-time education before 16 years old and those leaving after 16 (p = 0.33 and p = 0.93 respectively by Mann-Whitney).

### Section 3 – Verbal interpretation

**Figure 3:**
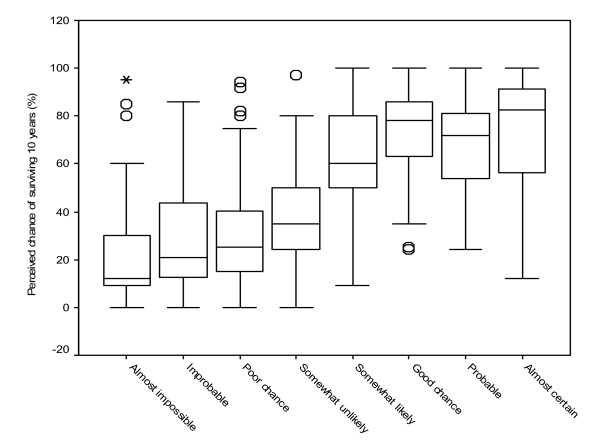
**Patients' perceptions of the equivalent percentage chance of being alive in 10 years, for eight different verbal descriptions.** (Median is represented by the vertical line, the interquartile range by the box and the range by the horizontal line. Outliers are illustrated by circles/asterisk).

For this section, assessing patients' interpretation of verbal statements relating to LE and survival probability, the responses are illustrated in figure 3. Overall the statements *Almost Certain *and *Good *chance of being alive in 10 years, were perceived by patients as equating to the highest percentage chance of being alive; mean (range) 74.5% (12–100) and 74.5% (24–100) respectively. *Almost impossible *was perceived as equating to the lowest percentage chance of being alive with a mean (range) of 21.5% (0–95). No statistically significant difference was noted between sexes or educational background.

### Section 4 – Visual interpretation

For this section, assessing patients' preferences for graphical display of LE and survival probability, the mean (SD) preference rating for the survival curve exceeded those of the three other graphs (table 1). 71% of patients ranked the survival curve as either their first or second most preferred graph, with 53% ranking the survival curve their first choice. 76% of patients ranked facial figures as their last choice. Mean (SD) preference rating for the LE line graph, on a 5 cm Likert scale marked 1 to 5 (1 = not at all, 5 = extremely), was 3.3 (1.1). Patients' reasons for rating the survival curve first choice included "clarity", "readily understood", "provides complete picture" and "shows survival not death". Reasons for rating the facial figure last choice included "muddled", "confusing", "childish" and "not enough detail". No statistically significant difference was noted between sexes or educational background.

**Table 1:** Mean preference ratings for each graphical format on a 5 cm Likert scale marked 1 to 5 (1 = not at all, 5 = extremely)

**Graphical Type**	**Overall (n = 120)**	**Male (n = 60)**	**Female (n = 60)**	**Completed Full-Time Education ≤ 16 yrs (n = 88)**	**Completed Full-Time Education >16 yrs (n = 32)**
**Survival Curve**	3.5 (1.2)	3.3(1.2)	3.6(1.1)	3.5(1.2)	3.5(1.2)
**Bar Graph**	3.1(1.1)	3.1(1.1)	3.2(1.2)	3.2(1.1)	2.9(1.2)
**Pie Chart**	3.0(1.3)	3.0(1.3)	3.1(1.3)	3.0(1.3)	3.0(1.3)
**Facial Figures**	1.8(1.3)	1.6(1.1)	2.0(1.4)	1.7(1.3)	2.0(1.3)

### Section 5 – Overall patient preferences

For this section, assessing patients' preferences for overall presentation format, 58.3% of patients ranked 'numbers & pictures' as being their first or second most preferred format for risk presentation, with 33% ranking it as their first choice. 'Pictures only' was not ranked in 83.3% of patients' top three choices, with only 3.3% ranking this their first choice. The use of 'numbers only' or 'words only' were each separately ranked first choice by 8.3% of patients.

Overall 64% of patients stated that they would want to know their predicted 10 year survival probability based on their medical history if it were available and 58% would want to know their predicted LE if it were available (table 2). 58% of patients stated that they would want to know both their predicted SP and LE, whilst 36% of patients would not want to know either SP or LE. 6% of patients would want to know their predicted SP but not their LE. Reasons for patients not wishing to know included "shocking", "fear", "feel time is running out", "might worry", "prefer to be ignorant" and "unpleasant to discuss date of death". No statistically significant difference was noted between sexes or educational background.

**Table 2:** Number of patients (percentage) that would wish to know their survival probability or predicted life expectancy if it were available in clinical practice

**Question**	**Response**	**Overall (n = 120)**	**Male (n = 60)**	**Female (n = 60)**	**Left Full-Time Education ≤ 16 yrs (n = 88)**	**Left Full-Time Education >16 yrs (n = 32)**
**10 yr Survival Probability**	**Yes**	77 (64%)	41 (68%)	36 (60%)	57 (65%)	20 (63%)
	**No**	43 (36%)	19 (32%)	24 (40%)	31 (35%)	12 (37%)
**Life Expectancy**	**Yes**	70 (58%)	38 (63%)	32 (53%)	51 (58%)	19 (59%)
	**No**	50 (42%)	22 (37%)	28 (47%)	37 (42%)	13 (41%)

## Discussion

This study has shown that no single format was preferred by patients when discussing LE and SP. In addition over one third of patients were unwilling to discuss their LE or SP if this information were available. In those patients willing to discuss such information, their interpretation was often inaccurate.

The findings of this study highlight the potential misinterpretation and variation in presentation format preferred by patients [7-9,11]. It has been suggested that patients should therefore be provided with a combination of numerical, verbal and graphical formats simultaneously [17,18]. In relation to LE and SP information, the current findings suggest that a combination of numerical and graphical formats is preferred, with the survival curve the preferred graph. The latter, however, has a tendency to 'positively frame' data and patients may be more influenced by the beginning and end points of the graph, compared with the interim medium-range data or point estimates [10,19]. The use of extended explanation (as in this study), however has been shown to reduce this problem [20].

Previous research suggests that about 15–30% of patients are unwilling to discuss their prognosis, particularly in relation to cancer [21-23]. This is thought to relate to a lack of understanding by patients and/or an unrealistic perception of their prognosis. In the present study 36% patients expressed an unwillingness to discuss their LE and SP if it were available. The two commonest reasons given by patients were 'fear' and 'worry'. It is unclear why 6% of patients would wish to know their SP, but not their LE. This may relate to a misinterpretation by patients of the LE as a definitive end point, i.e. more negatively framed, compared with SP. Whist LE itself is not an ethical or moral concept, when used in the decision-making process its significance becomes an important issue [24]. This clearly has implications for current surgical practice, in which predicted LE and SP can make the difference between patients receiving operative or non-operative treatment. Therefore in those patients willing to discuss issues relating to prognosis, it has been suggested that the content of such discussions be negotiated between doctor and patient, with information provided in a balanced manner and understanding verified [25]. Likewise, in those less willing to know, patients' reasons should be ascertained, an acknowledgement of their concerns made and where possible, alternative means of information provision sought [26]. The surgical community must remain sensitive to this issue, ensuring that patients are appropriately counselled before discussion. The provision of medical information leaflets, increased contact with nurse specialists to reinforce understanding and further education/training of clinicians may facilitate this process.

## Limitations

The present study did not provide patients with an individualised estimate of their own predicted LE or SP, due to the impracticality of calculation in a convenient survey time. In addition no assessment was made of how this information would have influenced real or hypothetical treatment choices. The use of mixed graphic types with both fixed and multiple time points may have led to framing effects influencing patients' preferences. The patient population studied were aged over 50 years and poorly educated; whilst these results may therefore not be generalisable to all patients, they relate to the commonest age group attending hospital for medical and surgical treatment.

## Conclusion

The discussion of LE and SP is an important component of decision-making and informed consent in surgical patients, since it can have a significant influence on the treatment options offered to patients. However whilst patients' understanding of this is often assumed by clinicians, the current results highlight the alarming variation in interpretation and preferences for presenting such information. In addition a proportion of patients may be unwilling to discuss their LE or SP. As a consequence clinicians must be sensitive to these issues. Greater education and training of doctors in the communication of risk, including clarification of effective patient understanding and a wider use of available resources, will ensure that patient-centred treatment decisions are made more appropriately.

## Competing interests

The authors declare that they have no competing interests.

## Authors' contributions

MGC was involved in conception, design, analysis and interpretation of data in addition to drafting first article. KPK involved in drafting article and revising critically. RPM involved in design of study and final approval of version to be published. All authors approved the final manuscript.

## Pre-publication history

The pre-publication history for this paper can be accessed here:


